# Structural Evolution of Global Soybean Trade Network and the Implications to China

**DOI:** 10.3390/foods12071550

**Published:** 2023-04-06

**Authors:** Min Wang, Dong Liu, Zhenxing Wang, Yuetan Li

**Affiliations:** College of Resources and Environment, University of Chinese Academy of Sciences, Beijing 100049, China

**Keywords:** soybean trade, complex network, structural evolution, targeted disruption, food security, China

## Abstract

China experiences a serious shortage of soybean supplies and relies heavily on international trade with high vulnerability and large uncertainty, which maybe sows food security risks. It is of great significance to analyze the structural evolution of the global soybean trade network and its implications to China for ensuring food security. This paper constructed a global soybean trade network (GSTN) and analyzed the structural evolutionary characteristics of GSTN from 2000 to 2020 using the complex network analysis method and simulated the impact of targeted destruction on China through scenario analysis. The results showed that GSTN was gradually complex exhibiting a small word and a scale-free network property. The global soybean exporter was dominated by some major soybean-producing countries in America. The US played an important role in maintaining GSTN’s robustness. China was the world’s largest soybean importer; unfortunately, its soybean imports relied heavily on a few countries, and the anti-interference ability of China’s soybean trade tended to decline. Therefore, China’s soybean trade was increasingly vulnerable to being tightly controlled by other countries when some uncertain factors occurred, such as trade frictions and changes in policy decisions from importing and exporting countries. The US and Brazil were key countries with significant soybean trade ties to China. To assess the impact of the two countries on China’s soybean trade, targeted destruction method was used through destroying them in the network. Targeted destruction scenario analysis indicated the two countries played important roles in the anti-interference ability of China’s soybean trade. Brazil played a positive role in China’s control of soybean trade flows, while the US did not. Some policies for China’s soybean production and international trade were proposed. A balance between the domestic production and import of soybean is needed. Optimizing the soybean trade import system and seeking more trade partners is crucial. Improving soybean self-sufficiency is the fundamental way to reduce the high-import dependence. The study provided some insights for coping with international market fluctuations and improving the sustainability of China’s soybean trade.

## 1. Introduction

Soybean is one of the important major food crops in China. It is not only the raw material for soy products but a commodity crop with industrial uses both in the livestock and edible oil sectors. Those sectors have grown rapidly in response to dietary shifts towards more animal protein (e.g., meat, egg, and milk) [1]. Accordingly, soybean consumption has risen sharply in recent years. China relies heavily on international soybean imports for a long time. The soybean imports increased from 10.4 million tons (Mt) in 2000 to 100.3 Mt in 2020 [2], and the import dependence increased from 46.2% to 83.6% [3]. It is estimated that by 2030, China’s soybean consumption will reach 141.7 Mt while the domestic production just will be 21.7 Mt [4]. Despite the Chinese government issuing a series of policies to support soybean production (such as the soybean revitalization plan), the situation of short supply is still severe, which may seriously threaten China’s food security. Analysis of the structural evolution of the global soybean trade network is necessary to understand how to ensure China’s food security.

The complex network method is an important tool to study the characteristics and dynamics of international trade systems using topological features, which was widely applied in public transportation [5,6], air quality [7], disease transmission [8,9], and natural resource flow [10,11,12]. In terms of grain trade [13,14], Srishti et al. [15] analyzed the impact of extreme weather on wheat trade by the complex network; Zhou et al. [16] built the global rice network and studied its evolution characteristics. Lu et al. [17] constructed different weighted and unweighted trade networks and analyzed the trade networks of soybean, soybean oil, and soybean meal using complex network analysis. Kou et al. [18] built the global soybean trade networks and analyzed the basic topological properties of the trade networks. Mendes et al. [19] studied the soybean trade of three major exporters with their top ten commercial partners and evaluated the interdependence between exporters and importers by the complex network method. The complex network has proven applicable to characterizing the patterns of international trade.

The international grain trade flow is affected by policy decisions [20], diplomatic relations [21], trade frictions [22], regional conflicts [23], natural disasters [24], and other factors. For example, Vietnam temporarily banned rice exports in 2020 to ensure domestic food security, triggering sharp fluctuations in global rice prices [25,26]. In 2018, China decided to impose a 25% tariff on US soybeans, resulting in the first drop in Chinese soybean imports in the past decade [27]. Influencing factors, such as the COVID-19 pandemic and China–US trade frictions, came up frequently in recent years. We expected those factors changed the structure of the global soybean trade network. We hypothesized that changes in global soybean trade would affect China’s soybean supply chains and the robustness of the trade network. Therefore, here we constructed a global soybean trade network (GSTN) and applied the complex network analysis method to analyze the spatio-temporal structural evolution characteristics of the global soybean trade from 2000 to 2020 and explore the impact on China’s soybean trade robustness and the implications to China. We tried to answer the following questions: (1) What structural changes took place in the GSTN during the past two decades? (2) What was the impact of the changes on China’s soybean trade? (3) What were the implications for China to respond to changes in the international market and promote the sustainability of soybean trade?

## 2. Materials and Methods

### 2.1. Complex Network Analysis

Weighted and directed global soybean trade network G = (V, E, W) is constructed in this paper. V represents a collection of countries or regions; E is the trade direction between countries, and W denotes the weight of edges connecting the exporting country to importing country. The basic topological properties of complex networks include network density, path length, network diameter, weighted degree, betweenness centrality, closeness centrality, clustering coefficient, and modularity. The brief meanings of these indicators are summarized in Table 1.

Network density (*ρ*) refers to the ratio between the actual number of edges *M* and the maximum possible number of edges in the network. It is used to measure the closeness of trade between countries. The value range of network density is [0, 1]. The closer the value is to 1, the closer the trade ties between countries. For a directed network, *N* is the number of nodes.

$$\rho =\frac{M}{N\left(N-1\right)}$$

Average path length (*L*) of the network is the average of the shortest distance between any two points, presenting the efficiency of trade between countries. $d_{ij}$ represents the distance between node (country) *i* and *j*, and higher average path length means higher transmissivity efficiency.

$$L=\frac{2}{N\left(N-1\right)}\overset{N}{\underset{i\geq j}{\sum }}d_{ij}$$

Network diameter (*Dia*) is defined as the maximum distance between any two nodes (countries). The value of *Dia* is proportional to the scale of the network and can be used as a measure of the network size.

$$Dia=\underset{ij}{\max}d_{ij}$$

Degree denotes a node’s total number of direct trade partners, i.e., the number of direct trade flows between countries or regions. Weighted degree ($C_{sum,i}$) expresses the total amount of trade between node *i* and other nodes. In the directed network, outdegree ($C_{out,i}$) and indegree ($C_{in,i}$) are the number received and sent by node *i*, corresponding to the actual amount of soybean export or import flows of country *i*.

$$C_{sum,i}=C_{out,i}+C_{in,i}$$

$$C_{out,i}=\overset{N}{\underset{j=1,i\neq j}{\sum }}W_{ij}$$

$$C_{in,i}=\overset{N}{\underset{j=1,i\neq j}{\sum }}W_{ji}$$

Betweenness centrality (BC_k_) indicates whether a node is in the shortest path (geodesic) of any two other nodes and shows the control of the node over the resource flows. $n_{ij}^{k}$ indicates the number of nodes *k* to pass through the geodesic between node *i* and *j*, and $g_{ij}$ indicates the number of geodesics between node *i* and node *j*. The points with higher betweenness centrality usually play a more important role in the network.

$$BC_{k}=\underset{j\neq i\neq k}{\sum }\frac{n_{ij}^{k}}{g_{ij}}$$

Closeness centrality (CC) is an indicator that measures the distance between a node and all other nodes in the network. It is used to consider the independence and resistance of nodes when they are under the influence of external factors. It shows the ability of a particular country to protect itself against trade risks from other countries, with the range of [0, 1]. The closer the value is to 1, the stronger the anti-interference ability of the country.

$$CC_{i}=\frac{N}{\sum_{i=1}^{N}d_{ij}}$$

Clustering coefficient (C) represents the ratio of the maximum number of connections between *i* and its neighbor nodes $K_{i}$ and the actual number of connections with neighboring nodes $\;E_{i}$. It is used to indicate the aggregation of nodes in the network and measure the connection strength between countries in the GSTN over the years with a value range of [0, 1]. Higher clustering coefficient means higher network connectivity.

$$CC_{i}=\frac{2E_{i}}{K_{i}\left(K_{i}-1\right)}$$

Modularity (*Q*) is used to measure the degree of division of the communities in a network and to compare the communities with the whole network.

$$Q=\frac{1}{2m}\underset{ij}{\sum }(A_{ij}-\frac{k_{i}k_{j}}{2m})\delta \left(C_{i},C_{j}\right)$$

$\delta \left(C_{i},C_{j}\right)$ is used to measure the unity of the community to which node *i* and node *j* belong. If node *i* and node *j* belong to the same community, $\delta \left(C_{i},C_{j}\right)$ = 1, otherwise $\delta \left(C_{i},C_{j}\right)$ = 0. $A_{ij}$ is any element of the adjacency matrix. If node *i* is connected to node *j*, $A_{ij}$ = 1, otherwise $A_{ij}$ = 0. *m* is the total number of edges and $k_{i}$, $k_{j}$ denote the degree of node *i* and node *j*, respectively. *Q* ranges from 0 to 1, and a higher value means a clear clustering structure of the network.

Hub value (Hub_p_) is calculated by Hyperlink-Induced Topic Search (HITS) algorithm, which is designed to rank webpages initially [28]. The calculation of the hub value is associated with the authority value Auth_p_ generated by running the algorithm. A node with a higher hub value than the other nodes plays a hub role in the network. The initial Hub_p_ and Auth_p_ of each node are set as 1 and updated in every iteration step until they are stable through the following formulas:

$$Auth\left(p\right)=\underset{q\in p_{to}}{\sum }hub\left(q\right)$$

$$Hub\left(p\right)=\underset{q\in p_{from}}{\sum }auth\left(q\right)$$

### 2.2. Robustness of the Complex Networks

Network robustness is defined as the resilience of a complex network to disruption. If the disruption of a node leads to a large-scale failure of the network, showing the node playing a key role (i.e., a hub). Network efficiency (E) is often used to evaluate network robustness, especially the connectivity between nodes and the overall efficiency [29]. Targeted disruptions of these nodes can demonstrate the impact on the efficiency of the entire network. By comparing the impact on the GSTN when the hubs are disrupted, it is possible to simulate the situation where the country is unable to trade in the event (trade frictions, wars, epidemics, etc.). It helps to find how the important countries in the GSTN affect the whole network and thus assess the impact on other countries. If network efficiency decreased when a country was missed from the GSTN, it indicated that the network robustness and anti-attack capability became weak.

$$E=\frac{1}{n\left(n-1\right)}\sum \frac{1}{d_{ij}}$$

### 2.3. Data Sources

Bilateral trade data for soybeans in this paper are from the FAOSTAT Detailed Trade Matrix database (www.fao.org/faostat, accessed on 15 May 2021). According to UN FAO Norms and the International Convention on the Harmonized Commodity Description and Coding System (HS) standard, the code for soybean is HS1201. In the FAO Trade Matrix data, each bilateral trade volume is reported by both the exporting and importing countries. Theoretically, the export volume reported by country A to country B should be equal to the import volume reported by country B from country A. However, the data is often not the same in reality. Considering import countries need to impose tariffs on imported goods, it makes the import data more precise. Through reviewing the data, we also found that the data recorded on the importers was significantly more complete than those recorded on the exporter. Therefore, data of soybean import quantity is used with five-year intervals selected (2000, 2005, 2010, 2015, and 2020) for analysis.

## 3. Results

### 3.1. Structural Evolution of the GSTN

#### 3.1.1. Basic Topological Properties of the GSTN

The global soybean trade volume grew from 48.1 Mt in 2000 to 165.0 Mt in 2020, with an increase of 2.43-fold and an average annual growth rate of 6.4%. With the growth of trade volume, the GSTN was gradually increasing and complex. Several basic topological properties of GSTN are shown in Table 2. The number of nodes (countries) involved in the trade rose substantially from 123 to 161 during 2000–2020, with the largest number of 161 in 2015. The fastest growth of participating countries occurred in 2010–2015, followed by a gradual stabilization after 2015. The number of trade flows (edges) between countries experienced continuous growth from 660 to 1010 with a 1.53-fold increase. The growth of the nodes and edges reflected the gradual increase in the participation of countries in the global soybean trade over the past two decades. The mean network density was 0.0384, with a maximum of 0.043 in 2010 and a minimum of 0.035 in 2005, indicating the GSTN tended to form stable trade flows and specific trade groups. The average degree and average clustering coefficient had an upward trend from 5.038 to 6.273 and from 0.261 to 0.342, respectively. The average weighting degree related to the volume of soybean trade also increased by 1.79-fold in 2020 compared to 2000. The average path length decreased with fluctuations from 2000 to 2020, presenting the improvement of the efficiency and the closeness of trade between countries.

**Figure 1:**
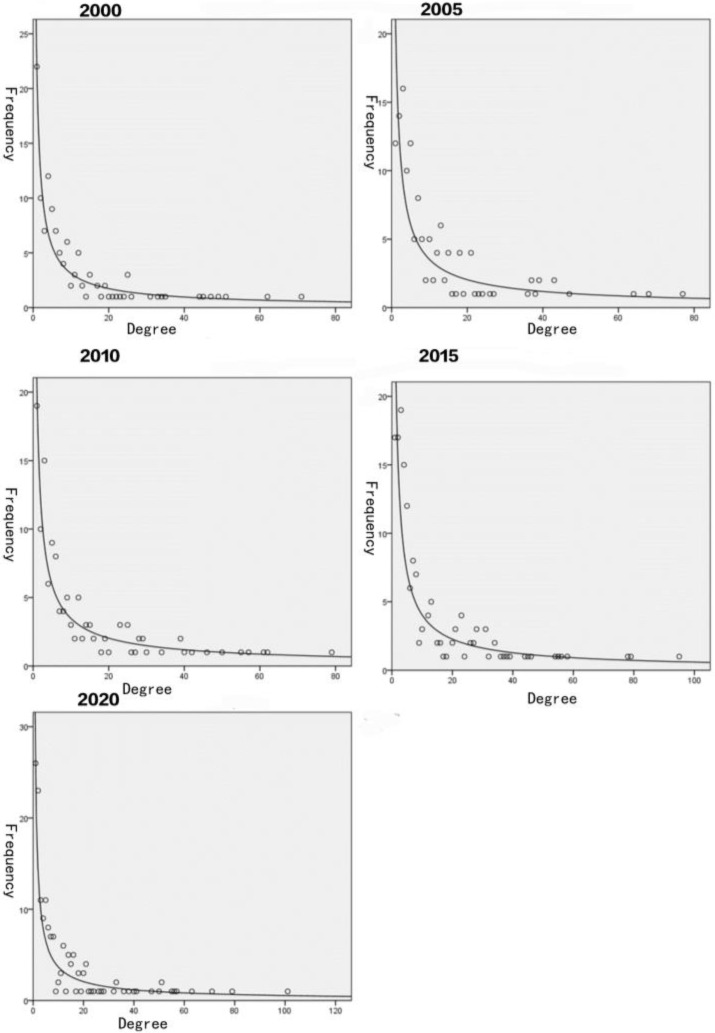
Power law distribution fitting of node degree of GSTN in 2000, 2005, 2010, 2015, and 2020 (data source: FAO). Note: degree (X-axis) is the number of direct trading partners of a country; frequency (Y-axis) is the number of countries with a certain degree.

Some characteristics of the GSTN were found based on the topological properties: (1) a small-world property. It is reflected by the low path length and the high clustering coefficient of the network, which enable any two countries in the network to connect through other countries with a higher trade efficiency. (2) A scale-free network property. It means severe heterogeneity and inhomogeneous distribution of degree. The node degree distribution of the GSTN have a power law feature, i.e., a few nodes have an extremely large number of connections, while most nodes have a small number of connections (Figure 1). In Figure 1, the degree of the node (i.e., the number of direct trading partners of a country) was the X-axis, and the frequency of the node’s degree (i.e., the number of countries with a certain degree) was the Y-axis. The node with a large number of connections can be called a hub playing a dominate role in the network to maintain the structure. Therefore, the scale-free network is highly fault tolerant because most nodes would not influence the normal function even if they were attacked. However, if the hub(s) was targeted, the network would become much less resistant to attack. Therefore, the hub(s) largely determines the robustness of the network.

#### 3.1.2. Community Analysis of the GSTN

**Figure 2:**
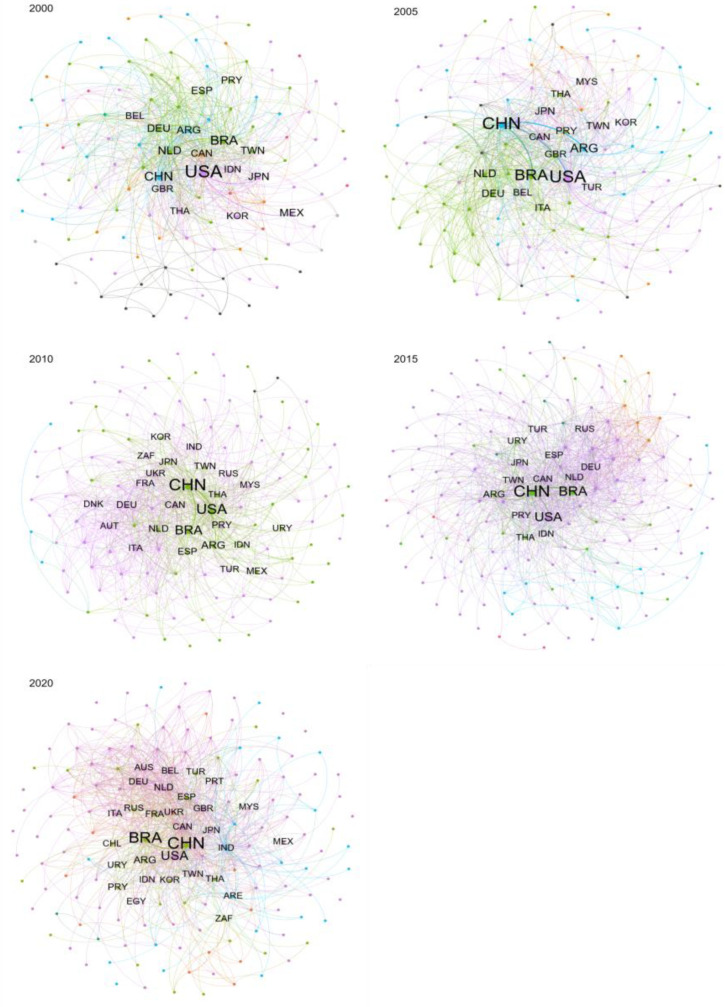
Global soybean trade network in 2000, 2005, 2010, 2015, and 2020 (data source: FAO). Note: nodes represent countries or regions, and their size are proportional to the trade volumes. The edges represent the weight of the soybean trade volume, and their widths are positively correlated to the bilateral trade volumes. Different colors of nodes and edges represent different communities in the GSTN. The countries in the same community had closer trade relationships. Trade relations between countries in the same community are much closer than countries in different communities.

The Louvain community division algorithm is often used to detect communities or clusters in large networks. It uses a hierarchical approach to partition nodes into communities by optimizing a modularity score that measures the density of links within communities. We employed the Louvain community division algorithm to distinguish the communities of GSTN in 2000, 2005, 2010, 2015, and 2020 (Figure 2). Compared to countries in different communities, the countries in the same community had closer trade relations with each other.

The community number of GSTN presented an upward trend, and the network structure became multipolar. The trade network could be roughly divided into two main communities in 2000, namely the Europe–America community and the Asia community. In 2005, the GSTN was composed of three communities: North America and parts of the Asia community, China and the South America community, and the Europe community. In 2010, GSTN can be briefly divided into two major communities: the America–East Asia community and the Europe–Africa–West Asia community. The largest community including 158 countries appeared in 2015: the Europe–Asia–North America–Oceania community, forming the GSTN along with the South America community. However, in 2020 the trade network evolved into four main communities, i.e., the Europe–America community, the Asia–South America community, the Africa–Middle East–South Asia community, and the Southern Europe–East Africa community. The modularity indicator oscillated throughout the study period (Table 2) indicating that the trade partner cooperation was constantly changing as the trade network became more complex.

#### 3.1.3. Changes of Importer and Exporter of the GSTN

**Figure 3:**
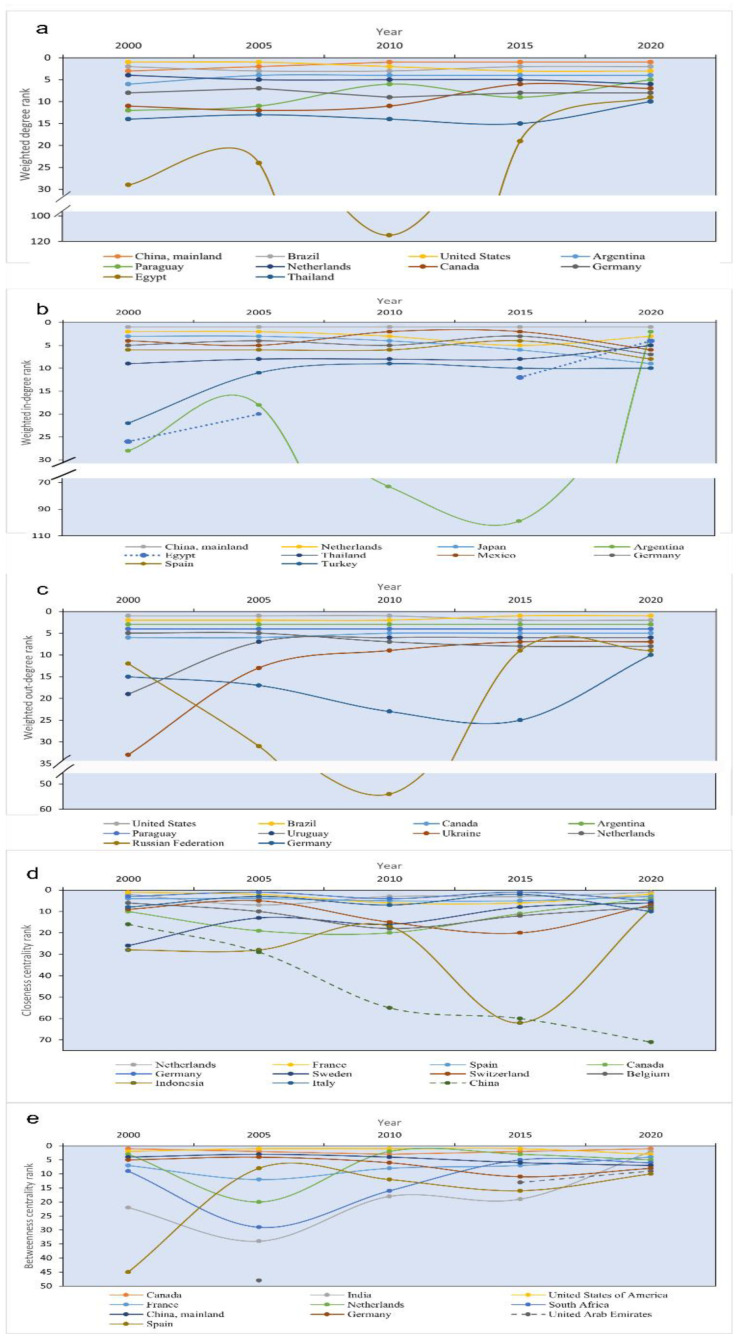
Dynamic rankings of the top-ranked countries (data source: FAO). (**a**) Weighted degree; (**b**) weighted indegree (due to the missing import data, Egypt was specifically indicated by dotted lines); (**c**) weighted outdegree; (**d**) closeness centrality; and (**e**) betweenness centrality.

In order to identify the importance and change of major import and export countries in global soybean trade, we showed the dynamic ranking of the top-ranked countries in terms of weighted degree, weighted indegree, and weighted outdegree during 2000–2020 (Figure 3). The US, Brazil, Argentina, China, Netherlands, and Germany were the major soybean trade countries and firmly remained in the top ten (Figure 3a). The US ranked the first place until 2005, after which China replaced the US in 2010 and remained for ten years. China was the major driver of global soybean trade over the last decades. For import countries, some Asian countries and European countries dominated the top ten (Figure 3b). China ranked first place since 2000, indicating that China was the world’s largest soybean importer in the world. Japan also was a large Asian food importer due to its mountainous terrain and limited resources with high indegree ranking. The rank of Thailand kept rising from 9th in 2000 to 5th in 2020. Additionally, Netherlands, Germany, and Spain were major soybean importers in Europe, firmly in the top ten. The global soybean export market reminded stable and dominated by some major soybean producers in South America and North America, such as the US, Brazil, Argentina, and Paraguay (Figure 3c). Except for Uruguay, which ranked 19th in 2000 and then rose to 7th in 2005, other South American countries, including Brazil, Argentina, and Paraguay were all in the top ten. The US was the world’s largest soybean exporter before 2015 and then was replaced by Brazil. Notably, Russia fluctuated significantly, ranking 12th place in 2000, 31st in 2005, 54th in 2010, and 9th place in 2015 and 2020. It was attributed to the continued and rising soybean export to China after 2012.

According to the ranking of closeness centrality (Figure 3d), the top ten countries were dominated by developed countries, including the US, France, Spain, Germany, Netherlands, and Italy. However, only the US was in the top 10 in 2020, while other developed countries were being replaced by developing countries. From the rank of betweenness centrality (Figure 3e), we could see the US steadily ranked among the top ten from 2000 to 2020, indicating the important role and the enormous voice in the GSTN.

### 3.2. Disruption Simulation of GSTN and the Impact on China

#### 3.2.1. Structural Changes of China’s Soybean Trade

**Figure 4:**
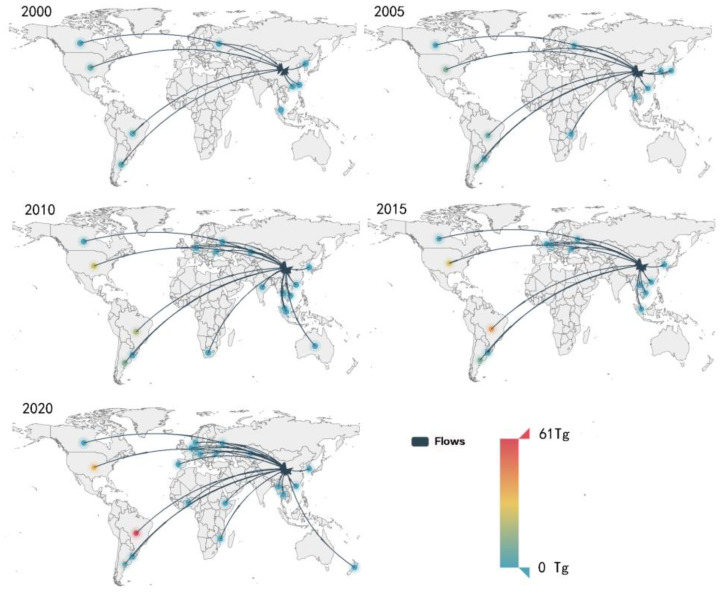
The main trade flows of China’s soybean imports in 2000, 2005, 2010, 2015, and 2020 (data source: FAO). Note: the figure only shows the trade flows greater than 10 tons.

From the analysis of Section 3.1.3, it was clear that China remained the largest soybean importer in the world during the last two decades. Driven by the domestic demand for soybean, China imported a huge amount of soybean; from 2000 to 2020, China’s soybean import quantity increased from 10.4 Mt to 100.3 Mt, increasing by 8.6-fold. With the complex networks, we analyzed the major trade flows associated with China. Figure 1 and Figure 4 demonstrated the community changes of China’s soybean trade and China’s main soybean importing countries from 2000 to 2020, respectively. In Figure 4, we only showed importers whose trade volume was greater than 10 tons. The node color represented the trade volume. The redder the color, the larger the trade volume.

**Figure 5:**
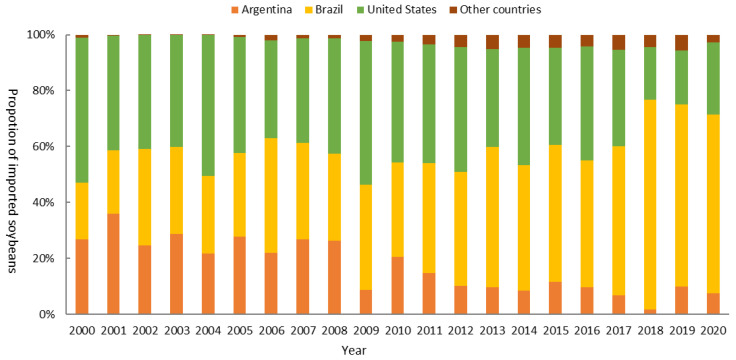
Proportion of China’s soybean imports from 2000 to 2020 (data source: FAO).

The largest community China participated in occurred in 2000 when 54 countries traded closely with China, such as the US, Argentina, and Japan. In 2005, the community China joined included 18 countries, mostly from Asia and Africa. Around 2010, China entered the community with the US and Brazil, which was attributed to the large trade volume with them. Then, Brazil’s exports to China continued to increase after its share surpassed that of the US in 2013 (Figure 5). China withdrew from the US-led community because of the more trade ties with South American countries, such as Brazil and Argentina (Figure 1). In 2020, the trade network evolved into four main communities, i.e., the Europe–America community, the Asia–South America community, the Africa–Middle East–South Asia community, and the Southern Europe–East Africa community. China joined the Asia–South America community with 30 other countries, including Brazil, Paraguay, etc.

Closeness centrality can reflect the node’s anti-interference ability to cope with external changes and disruptions. The rank of China’s closeness centrality continued to decline, falling from 16th in 2000 to 71st in 2020 (Figure 3). It indicated that the anti-interference ability of China’s soybean trade decreased during the past two decades. China’s soybean trade was increasingly vulnerable to being tightly controlled by other countries when some uncertain factors occurred, such as trade frictions and changes in policy decisions from importing and exporting countries. Although the communities and China’s trade countries changed during 2000–2020, Brazil, the US, and Argentina were important trade partners in recent years (Figure 4). The soybean import quantities from these three countries accounted for over 95% of the total to China. Especially, Brazil and the US kept a grip on China’s soybeans, accounting for 90% of import quantities in 2020 (Figure 5). The US was China’s largest soybean importer until 2013, where the import share declined from 52.0% in 2000 to 35.1% in 2013. Brazil began to displace the US as China’s largest supplier of soybeans in 2013. By 2020, the import quantity from Brazil reached 64.3 Mt, accounting for 64.2% of China’s total soybean import volume. The US and Brazil had a notable impact on China’s soybean trade.

#### 3.2.2. Targeted Disruptions of GSTN and the Impact on China’s Soybean Trade Market

As described in Section 3.1.1, GSTN exhibited a scale-free property, and its structure and robustness were dominated by several highly connected hub countries. To identify the critical countries of GSTN, we calculated the hub value of each country in the network and selected the important hub countries that had significant trade relations with China. Then, through excluding the important hub countries to China, evaluated the network robustness and the impact on China’s soybean trade market using targeted destruction method (i.e., destroying the targeted countries in the trade network to assess the influence of them on specific country or the whole network). By calculating the hub value of the GSTN in 2020, we found that most of the EU countries had high hub values due to the small trade volume with China; however, they had a limited impact on China. Also, we found that the node representing the US was a hub with more trade flows to China, while Brazil became a major soybean trading country with China in recent years, albeit with a slightly lower hub value. Therefore, the US and Brazil, two hubs with enormous influences on China’s trade, were targeted to destruct; the impact of targeted destruction on China’s trade was assessed by comparing the network topological properties before and after the destruction based on 2020. In this paper, three destruction scenarios were conducted for analysis: only the hub representing Brazil excluded (Scenario 1); only the hub representing the US excluded (Scenario 2); both hubs excluded simultaneously (Scenario 3). The network efficiency was almost unchanged (0.305) in Scenario 1, showing Brazil had a limited impact on the GSTN’s robustness. When only the node representing the US was excluded (Scenario 2), the trade network efficiency reduced to 0.290. It demonstrated that the US played a pivotal role in maintaining the robustness of GSTN. In Scenario 3, the network efficiency declined to 0.286. GSTN’s robustness weaken further when Brazil and the US withdrew from the trade network. However, there were only subtle differences between Scenario 2 and Scenario 3.

Compared with the pre-disruption, the closeness centrality of China showed a decreasing trend in all three scenarios (Table 3). We noted that the closeness centrality of China decreased from 0.3934 to approximately 0.37 with either Brazil or the US excluded (Scenario 1 and 2), indicating the two countries played equal roles in the anti-interference ability of China’s soybean trade, although Brazil had a limited impact on GSTN’s robustness. In Scenario 3, the closeness centrality of China decreased to 0.3686. It showed the anti-interference ability of China’s soybean trade was significantly weakened and more susceptible to external influences when Brazil and the US were excluded simultaneously. Therefore, Brazil and the US played important roles in the anti-interference ability of China’s soybean trade.

The betweenness centrality of China showed a different trend after the destruction (Table 3). The betweenness centrality decreases from 928.250 to 895.334 in Scenario 1, meaning China’s ability to control the soybean trade flows weakened. On the contrary, the betweenness centrality of China increased significantly to 1043.256 in Scenario 2. It demonstrated that in this case, China’s ability to control the soybean trade flows enhanced after the destruction of the node representing the US. In Scenario 3, China’s betweenness centrality increased and, therefore, the same as Scenario 2, China’s ability to control soybean trade flow presented an increasing trend. The results showed that the existence of Brazil was more favorable for China to control the soybean trade, while the US was not. The US was more important to China than Brazil in terms of controlling soybean trade flow.

The US was the largest soybean trade partner before 2013, and the share of the US soybean was 52.0% in 2000; however, the share fell to 35.1% in 2013, then Brazil overtook the US as China’s largest source of soybeans. Due to the Sino–US trade friction, the share of the US was at an all-time low in 2018 of only 18.9% of China’s total soybean imports. To bridge the gap, China turned to South American countries, such as Brazil and Argentina. In 2020, China’s soybean importing quantity reached 100.3 Mt, more than that of 2017, and the majority of importing was from Brazil and Argentina. It indicated that on the one hand, Brazil and Argentina played a crucial role in the soybean trade of China; on the other hand, China had the ability to reduce the trade risk and meet importing demand when the US decreased trade volume.

## 4. Discussion and Policy Implications

Trade network analysis could help explain the trade characteristics and dynamics of a country and further assess the relationship between domestic production and international trade and, therefore, provide suggestions for production and trade. We discussed the implications of the structural evolution of the global soybean trade to China’s soybean production and international trade.

### 4.1. Balancing Import and Domestic Production of Soybean

**Figure 6:**
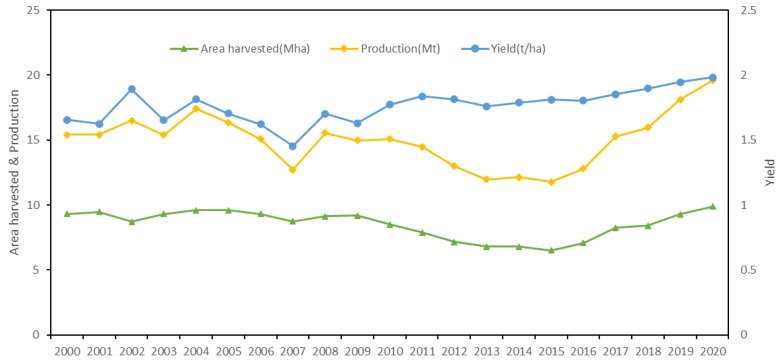
Area harvested, output, and yield of China’s soybeans production from 2000 to 2020 (data source: FAO).

Only when food production is sustained can a country maintain its international competitiveness for a long time. Compared with industrial mechanization countries, China’s soybean production is still dominated by household farming making it difficult to achieve large-scale production and economic effects [30]. Although some measures were conducted to stimulate domestic soybean production, the soybean yield and the harvest area have not increased significantly (Figure 6). It seems unrealistic for China to rely solely on domestic production to meet the demand in the short term; therefore, international trade is the way to solve the domestic supply shortage. Some studies showed that, for China, importing soybean could consume less water and arable land, which was conducive for reducing pressure on important resources [31]. It is noted that every coin has two sides. On the one hand, importing soybean is helpful to stabilize the domestic soybean market and save water and arable land resources; on the other hand, high import dependence (over 80%) not only sows food security risks but the relatively low price of foreign soybean also depresses the domestic soybean, further harming Chinese farmers’ willingness to plant soybean and thus creating a vicious circle. Hence, it is crucial for China to seek a balance between the domestic production and import of soybean.

### 4.2. Seeking More Soybean Trade Partners

Based on targeted disruptions and scenario analysis, we found China’s soybean import was vulnerable to be controlled by the leading trade countries (i.e., the US and Brazil) in GSTN. The share of China’s soybean imports from the US and Brazil accounted for over 85% in recent years. High dependence on foreign soybeans leads to large uncertainty for China’s soybean market when unexpected events arise. Nowadays, the international trade situation is a real roller coaster. During the trade friction between China and the US, China imposes a 25% tariff on imported US-made soybean [32], meaning Chinese soybean importers would have to pay more if continuing to trade with the US. The Brazilian Ministry of Economy showed that in 2020, Brazil’s soybean price rose as the stock declined and domestic supply became tight [33]. Those factors have led to the uncertainty in China’s soybean market [34]. Additionally, since the outbreak of the COVID-19 pandemic, some countries, such as Brazil, the US, and Argentina, have taken a series of measures to reduce their soybean exports to ensure adequate domestic food supplies. The rising cost, reduced supply, bad transportation, etc. resulted in the shrink of China’s soybean import [35]. In response to the international supply shortage, the Chinese government developed a series of measures to stimulate domestic soybean production. China’s soybean revitalization plan was released in 2019. The goal was to increase the soybean planting area by 10 million mu (666.7 thousand hm^2^) at the end of 2019 and to increase the sown area to 150 million mu (10 million hm^2^) by 2020. However, acreage increased only slightly in 2020 and actually decreased by 15% in 2021 [3]. Hence, the implementation of the policy cannot fill the gap in the short term. Pursuing imports is still an effective way to solve the problem. In order to improve the anti-interference ability in international soybean trade, it is necessary and crucial to optimize the soybean trade import system and seek more trade partners in the future. Our study showed that besides the US and Brazil, Paraguay, Argentina, and Uruguay are countries with high soybean yields and large export volumes. These are the potential countries for China to expand its importers. Furthermore, actively exploring work with Belt and Road Initiative countries is also an important option to alleviate the problem of soybean import. China has increased imports from Belt and Road Initiative countries, such as Russia, Ukraine, and Kazakhstan, in the recent years. In 2010, China imported only 678 tons of soybean from Russia, and the import quantity continued to rise to 693,000 tons in 2020 [3] accounting for 63.0% of Russia’s total soybean exports. Ukraine and Kazakhstan became new soybean trade partners in China’s soybean market in 2016 and 2017, respectively. The volume of trade in these new sources of soybeans is still small compared with Brazil, the US, and Argentina, the main sources of China’s soybeans trade, but it is certainly a useful complement to expanding importing countries.

Signing trade agreements is conducive to promoting the growth and development of trade. Some measures, such as cutting tariffs and granting most-favored-nation treatment, are used to create good trade relations among countries. In 2001, China joined the World Trade Organization and made some commitments in agricultural trade, including not restricting soybean imports and reducing import tariffs. Since the Second Amendment to the Asia-Pacific Trade Agreement came into effect in 2018, China has cut soybean import tariffs to zero for some partners to counter the risk of insufficient soybean imports. Lowering soybean tariffs to reduce the import cost, increasing the quantity of soybean imports, and promoting import source diversification are important advantages for China to sign trade agreements. However, we should also note that uncertainties in the international market will bring volatility and risks to the Chinese market. If a flood of low-priced foreign goods enters the domestic market, domestic industries will be severely affected. Currently, China’s soybean supply is monopolized by several transnational companies, which seriously threatens the safety of China’s soybean industry [36]. Therefore, signing a trade agreement means both risks and challenges, but a harmonious and stable market environment is crucial for the Chinese and even the international market.

### 4.3. Improving Domestic Soybean Production and Soybean Self-Sufficiency

Improving soybean self-sufficiency is the fundamental way to cope with international market fluctuations and reduce high import dependence. To strengthen the planting area and yield is the primary measure. Some studies pointed out that profit was the greatest factor affecting grain production [37]. Statistics showed in 2019 that the average price and total cost of soybean in China were 3.75 RMB (US$ 0.54)/kg and 5.23 RMB (US$ 0.76)/kg, respectively, with a profit of −1.48 RMB (US$ −0.22)/kg, lower than the average profit of the three main crops (rice, maize, and wheat) [38]. The low profit harms the farmers’ willingness to plant soybean. Moreover, the low yield is another factor hindering the increase of soybean production. In 2020, the global highest yield of soybean was 3.43 t/ha in the US, and the world average yield was 2.86 t/ha, while China’s yield was only 1.98 t/ha [3]. Considering that profit is a key factor for farmers to determine which crop to plant, some measures should be implemented to arouse the farmers’ planting enthusiasm, such as raising the purchase price of soybean, improving subsidy policies, and income insurance policies for soybean producers. Additionally, varieties and planting methods and techniques are also important factors to improve soybean production. It is necessary to invest more in scientific research to breed high-yield soybean varieties and explore suitable planting methods and techniques, such as intercropping with other crops, grain, bean rotation, and so on.

## 5. Conclusions

Using the complex network analysis method, this paper analyzed the structural evolutionary characteristics of the global soybean trade and simulated the impact of targeted destruction on China through scenario analysis and finally discussed the implications to China’s soybean production and international trade. The results showed that the GSTN was gradually increasing and complex for the last two decades, exhibiting a small-world and a scale-free network property. With time elapsed, the GSTN became multipolar from two to four communities. The global soybean exporter was dominated by some major soybean production countries, such as the US, Brazil, and Paraguay. The US played a pivotal role in maintaining the GSTN’s robustness and had an enormous voice in global soybean trade. China was the world’s largest soybean importer; unfortunately, its soybean trade was increasingly vulnerable to being tightly controlled by other countries, especially the US and Brazil. In terms of controlling soybean trade flow, the existence of Brazil played a positive role to China, while the US did not. Although it seems unrealistic for China to rely solely on domestic production to meet the demand in the short term, a balance between domestic production and the import of soybean should be taken into consideration to reduce the high import dependence. Optimizing the soybean trade import system and seeking more trade partners are necessary and crucial for China. Measures can be taken to increase the acreage and yield to improve soybean production, such as raising the purchase price of soybean, improving subsidy policies and income insurance policies for soybean producers, breeding high-yield varieties, and exploring suitable planting methods and techniques. The study provided some insights for coping with international market fluctuations and improving the sustainable development of China’s soybean trade.

This study has its limitations. The period 2000–2020 was determined in this paper considering the frequent emergencies (such as pandemics, trade frictions, and regional conflicts) in recent years. However, it cannot completely reflect the whole picture of the changes in global and China’s soybean trade. A longer time series analysis with more data is necessary for future research. Multiple scenarios need to be included in the scenario analysis of targeted disruptions to simulate the impact on China. Additionally, the integrated telecoupling analysis for the relationship between soybean trade and food security is also a focus in our future research direction.

## Figures and Tables

**Table 1:** Summary of the basic topological properties of complex networks.

Index	Meaning
Network density	The closeness of the trade flows between each country.
Average path length	The efficiency of trade between countries.
Network diameter	The trade network size.
Degree	The number of direct trade flows between countries.
Weighted degree	The total amount of trade between a country and other countries.
Weighted outdegree Weighted indegree	The actual amount of soybean export or import flows of a country.
Betweenness centrality	The ability of a country to control over the trade flows.
Closeness centrality	The anti-interference ability of a country under the influence of external factors.
Clustering coefficient	The aggregation of countries in the trade network.
Modularity	The degree of division of the communities in a trade network.
Hub value	A country playing a hub or key role in the trade network or not.

**Table 2:** Topology characters of the global soybean trade network (data source: results from this study).

Year	Average Degree	Average Weighted Degree	Network Diameter	Graph Density	Average Clustering Coefficient	Average Path Length	Nodes	Edges	Modularity
2000	5.038	367,500.7	8	0.039	0.261	3.029	123	660	0.180
2005	5.122	447,186.0	8	0.035	0.253	2.890	135	758	0.342
2010	5.781	684,465.6	8	0.043	0.283	3.058	129	792	0.111
2015	6.012	777,411.7	7	0.036	0.326	2.822	158	998	0.319
2020	6.273	1,024,798.0	7	0.039	0.342	2.806	161	1010	0.165

**Table 3:** Basic topological properties of the GSTN after targeted destruction (data source: results from this study).

Scenario	Closeness Centrality	Betweenness Centrality	Average Path Length	Network Efficiency
Scenario 1—Brazil excluded	0.374	895.334	2.814	0.305
Scenario 2—US excluded	0.372	1043.256	2.850	0.290
Scenario 3—both Brazil and US excluded	0.369	994.713	2.819	0.286
Benchmark year—2020	0.393	928.250	2.806	0.302

Note: closeness centrality and betweenness centrality refer to China. Average path length and network efficiency refer to the GSTN.

## Data Availability

The data used in this paper can be accessed freely from online sources.
